# Current concerns about mental health in Bangladesh

**DOI:** 10.1192/bji.2021.43

**Published:** 2021-11

**Authors:** David Skuse

**Affiliations:** Professor of Behavioural and Brain Sciences, Division of Population, Policy and Practice, UCL Great Ormond Street Institute of Child Health, London, UK. Email: d.skuse@ucl.ac.uk

## Abstract

This month's issue of *BJPsych International* focuses on Bangladesh, one of the most densely populated countries in the world and geographically vulnerable to a wide range of natural disasters. Mental health has been deteriorating since the COVID-19 crisis, but few psychiatrists and clinical psychologists are available to manage the consequences.

Fifteen years have passed since we last published a country profile on Bangladesh in *BJPsych International*, so it is of great interest to find out how that country's psychiatric services have developed since then. In this issue we include several articles that will bring us up to date. Back in 2006, Bangladesh had only 73 psychiatrists in a country of 123 million people; just three of them had trained in child mental health.^1^ Since then, the population has grown by about one-third, indicating that a high proportion of the population is now under 15 years of age. According to the updated country profile by Tasdik Hasan and colleagues, mental health problems in both adults and children are at unprecedently high levels.^2,3^ The number of psychiatrists available to provide care to 162 million people has increased to 210 since 2005, but resources devoted to mental health services remain stubbornly low.^1^ The COVID crisis has made the situation even worse; in particular, there are concerns about the rapidly rising incidence of child mental problems, widely expressed on internet forums.

## New mental health legislation

Hasan et al provide a set of priorities for research and service development, but note that the World Health Organization's (WHO's) Mental Health Gap Action Programme (mhGAP) has yet to be implemented widely in Bangladesh.^2^ One promising development has been the provision of a new set of mental health laws, summarised by Mohammad Karim and Sabuj Shaikh.^4^ In 2018, the colonial era Lunacy Act of 1912 was replaced by a new Mental Health Act. One provision of the Act, which may lead to an improvement in currently inadequate government-run public health facilities (especially those outside urban areas), aims to support the development of separate mental health units in medical college and district-level government hospitals. Also, in the future, private entities will be able to establish rehabilitation centres.

## Prison psychiatry

Whether the 2018 Act will improve the fate of prisoners with mental disorders remains to be seen. Al Aditya Khan and colleagues review mental health services in the prisons of Bangladesh.^5^ There are no formal psychiatric services available to prisoners provided by the government, although some prisons have third-sector support for prison staff and inmates, aimed at improving employment chances on release and dealing with the problem of drug addiction. Many of the issues discussed would apply equally to prisoners in the UK, where drug misuse and mental health problems are rife. Interestingly, in Bangladesh psychiatrists do not visit prisons, and mentally ill prisoners are brought by staff to attend appointments elsewhere. The authors draw attention to the need for a comprehensive review of prisoner mental health using objective screening methodologies, and suggest that incarceration provides an opportunity to provide therapeutic interventions that may not be available elsewhere.

## The future of service provision

Finally, Faruq Alam and co-authors discuss how the provision of mental health services in Bangladesh could be improved in the future.^6^ This article also draws attention to the poor mental health of many transient Bangladeshi immigrants to the UK, as well as those working in the Persian Gulf states. The country has had to cope with more than its fair share of natural disasters and other stressors, not least of which has been the massive influx of refugees from Myanmar in the past few years. In some ways, these refugees have been better served than the indigenous population because they have received intervention from the WHO mhGAP initiative. Apparently, the consequent provision of psychiatrists and psychological support has caused local resentment because the immigrants are seen as competitors for scarce public service resources. Since the adoption of the country's new Mental Health Act of 2018, there is hope that fresh budgeting and funding will be used productively to improve services nationally, especially in rural areas. Improvements in crisis management are also a priority, given the scale and frequency of natural disasters in this part of the world.

## References

[1] Karim M, Shaheed F, Paul S. Psychiatry in Bangladesh. Int Psychiatry 2006; 3(3): 16–8. PMC6734680 31507854

[2] Hasan MT, Anwar T, Christopher E, Hossain S, Hossain MM, Koly KN, The current state of mental healthcare in Bangladesh: part 1 – an updated country profile. BJPsych Int 2021; 18: 78–82. 10.1192/bji.2021.41 PMC8554893 34747942

[3] Hasan MT, Anwar T, Christopher E, Hossain S, Hossain MM, Koly KN, The current state of mental healthcare in Bangladesh: part 2 – setting priorities. BJPsych Int 2021; 18: 82–85. 10.1192/bji.2021.42 PMC8554892 34747940

[4] Karim ME, Shaikh S. Newly enacted mental health law in Bangladesh. BJPsych Int [Epub ahead of print] 1 Feb 2021. Available from: 10.1192/bji.2021.1. PMC8554921 34748619

[5] Khan AA, Ryland H, Pathan T, Ahmed HU, Hussain A, Forrester A. Mental health services in the prisons of Bangladesh. BJPsych Int [Epub ahead of print] 26 Jul 2021. Available from: 10.1192/bji.2021.34. PMC8554923 34747941

[6] Alam F, Hossain R, Ahmed HU, Alam MT, Sarkar M, Halbreich U. Stressors and mental health in Bangladesh: current situation and future hopes. BJPsych Int [Epub ahead of print] 10 Dec 2020. Available from: 10.1192/bji.2020.57. PMC8554941 34747936

